# Koch's Remedy in Relation Specially to Throat Consumption

**Published:** 1891-06

**Authors:** 


*Koch's Remedy in Relation specially to Throat Consumption.* By LENNOX BROWNE, F.R.C.S. Ed. London: BAILLIÈRE, TINDALL, AND COX. 1891.

This handsome little book is one amongst the immense number of articles and pamphlets that have appeared during the present year, bearing on the action of tuberculin in cases of tuberculosis of the upper respiratory tract.

When it left the writer's desk in January, 1891, he had but recently returned from Berlin, and we can, therefore, excuse the optimistic tone of his work. We do not believe that increased experience bears out his statement that "phthisis in the beginning can be cured with certainty by this remedy." We hope that a definite proportion of all early cases may be cured, but we fear that "certainty" is not the correct word to use in this connection.

On the whole, the various points as regards dosage, selection of cases, treatment during reaction, &c., are well discussed; but we are all learners in this school, and much that was written in the beginning of this year may be found to be valueless by the end of it. As one instance of the way in which those who may be looked upon as authorities in Koch's remedy have altered their dosage, we may cite Prof. P. Guttmann, who suggested 1 mg. as the initial dose for adults in December last, and now recommends $\frac{1}{10}$ mg. as a proper quantity to administer at first.

The treatise is worth reading, for the description it gives of throat consumption, lupus, &c.; and it goes without saying that it is well illustrated and turned out of hand. It cannot, however, be regarded as an authoritative statement of the uses and value of tuberculin.

